# Uniportal thoracoscopic suture fixation of an endobronchial spigot for postoperative bronchial fistula

**DOI:** 10.1016/j.rmcr.2026.102489

**Published:** 2026-08-27

**Authors:** Gaku Yamaguchi, Haruka Kanai, Atsushi Morio

**Affiliations:** a International University of Health and Welfare, Ichikawa Hospital Division of Thoracic Surgery, Chiba, Japan; b Thoracic Surgery Department, Noda General Hospital, Chiba, Japan

**Keywords:** Bronchial fistula, Endobronchial spigot, Uniportal thoracoscopy, Postoperative complication

## Abstract

**Background:**

Postoperative bronchial fistula is a serious complication after lung resection, and when empyema develops, bronchoscopic placement of an Endobronchial Watanabe Spigot (EWS) is often attempted. However, in large postoperative fistulas, the EWS cannot be securely fixed and frequently migrates, rendering it ineffective. No established method exists to stabilize the EWS in such cases.

**Case report:**

A man in his 70s developed a persistent bronchial fistula after left lower lobectomy and lingular resection for metastatic lung cancer. An L-size EWS was placed bronchoscopically, but he developed empyema. Although the infection was controlled with antibiotics, the device repeatedly migrated into the pleural cavity, and the air leak persisted.

**Treatment:**

Using a single-port thoracoscopic approach, the fistula and the dislodged EWS were directly visualized. Because the bronchial wall was fragile, suturing the bronchial wall alone carried a risk of rupture. The EWS was fixed to the stump by passing sutures through the bronchial wall, the spigot, and the opposite bronchial wall. After a leak test, no residual air leak was detected.

**Results:**

The chest drain was removed on postoperative day 3. Bronchoscopy at 6 months confirmed complete closure of the fistula. The patient later died from recurrent colorectal cancer without recurrence of empyema or air leakage.

**Conclusion:**

Thoracoscopic suture fixation of an EWS is a minimally invasive and effective option for stabilizing the device in large postoperative bronchial fistulas where bronchoscopic placement alone is insufficient. This technique may be useful when direct stump closure is difficult due to tissue fragility.

## Highlights

• Large postoperative bronchial fistula treated with thoracoscopic EWS fixation.
• Repeated EWS migration prevented by direct suture fixation under uniportal VATS.
• Suture passed through bronchial wall, spigot, and opposite wall for stability.
• Technique avoided bronchial tearing in fragile infected tissue.
• Complete fistula closure achieved with early recovery and minimal invasiveness.

## 1 Introduction

Postoperative bronchial fistula is one of the most serious complications following lung resection. Because drainage alone is often insufficient, Clagett thoracotomy is frequently selected as the treatment of choice. However, Clagett thoracotomy imposes a substantial burden on both patients and healthcare providers and prolongs the overall treatment course. Various techniques, such as omental packing and continuous suction, have therefore been attempted to close bronchial fistulas or shorten the time to closure [1].

The Endobronchial Watanabe Spigot (EWS) is a silicone bronchial occlusion device widely used for persistent air leaks and postoperative fistulas [2,3]. By sealing the bronchial side of the fistula, bronchoscopic EWS placement prevents retrograde airflow into the airway, thereby allowing negative-pressure drainage of the empyema cavity to be maintained. However, the EWS is ineffective for fistulas larger than the device itself, and such cases are not amenable to bronchoscopic placement alone.

Here, we report a case in which a repeatedly dislodged postoperative EWS was successfully stabilized by directly suturing the device to the fistula from within the thoracic cavity using a single-port approach. This technique enabled secure closure of a large postoperative bronchial fistula that could not be controlled by bronchoscopic placement alone.

This study was approved by the Institutional Review Board of the International University of Health and Welfare, Ichikawa Hospital (Protocol No. 18-Ic-77-②; approved on March 1, 2023). Written informed consent for publication was obtained from the patient.

## 2 Case presentation

**Fig. 1:**
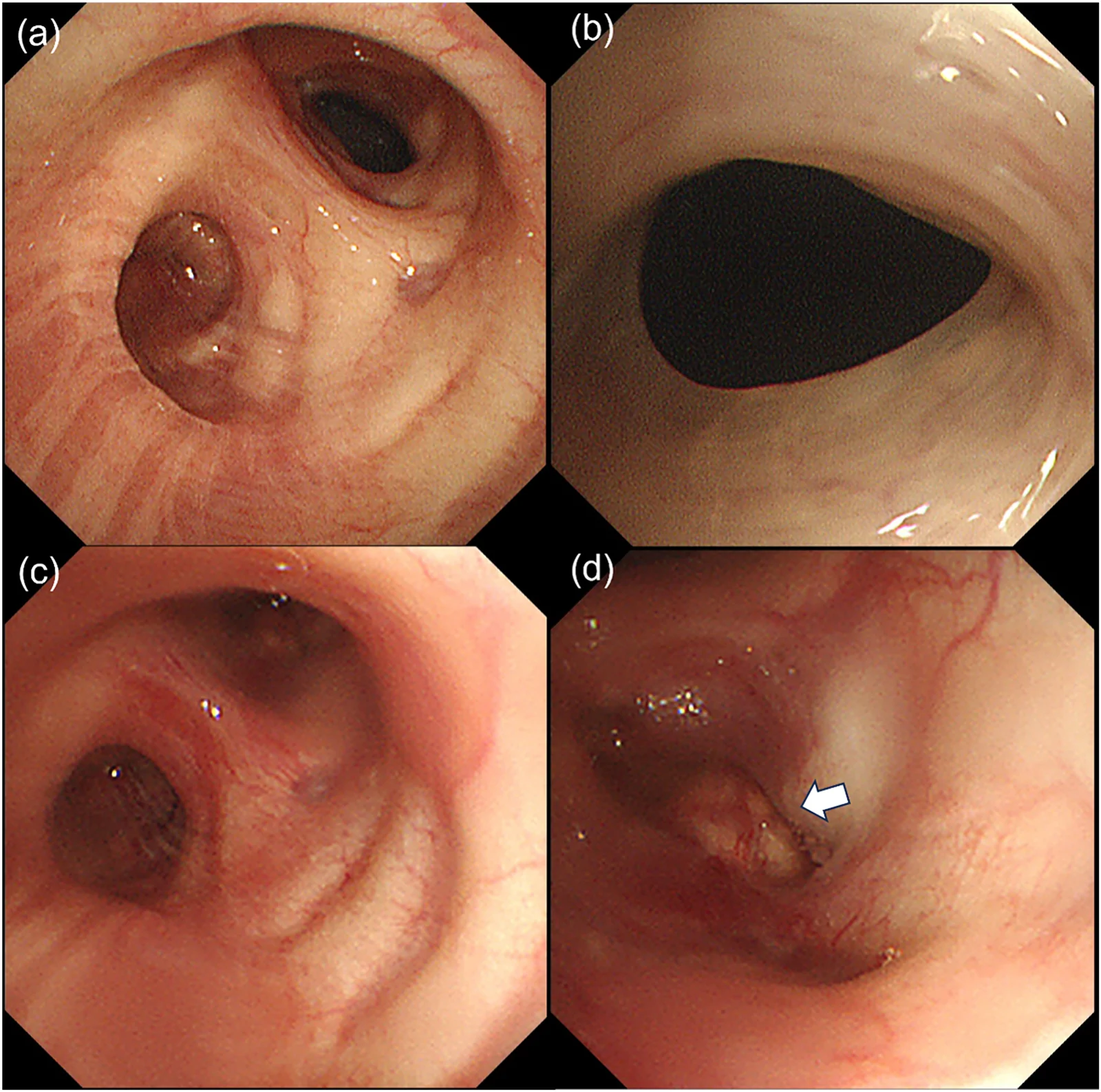
**Bronchial fistula before and after treatment**. (a) Distant view of the preoperative bronchial fistula. (b) Close-up view of the fistula. (c) Distant postoperative bronchial appearance at 6 months. (d) Close-up view showing the fistula occluded by the spigot and surrounding granuloma (arrow).

A man in his 70s with a history of kidney and colon cancer surgery was referred to our hospital with a diagnosis of metastatic lung tumor. A left lower lobectomy and lingular segmentectomy were performed, but a postoperative pulmonary fistula persisted, and an EWS was inserted into the upper division bronchus. He subsequently developed empyema and was treated with antibiotics. Although the cultures became negative, a new fistula appeared in the lingular segment. An EWS was inserted transbronchially into the fistula, but even an L-sized EWS repeatedly migrated into the pleural cavity. Because bronchoscopic fixation was not feasible and the bronchial wall appeared fragile, thoracoscopic surgery under general anesthesia was planned to secure the EWS by suturing (Fig. 1).

## 3 Intervention

**Fig. 2:**
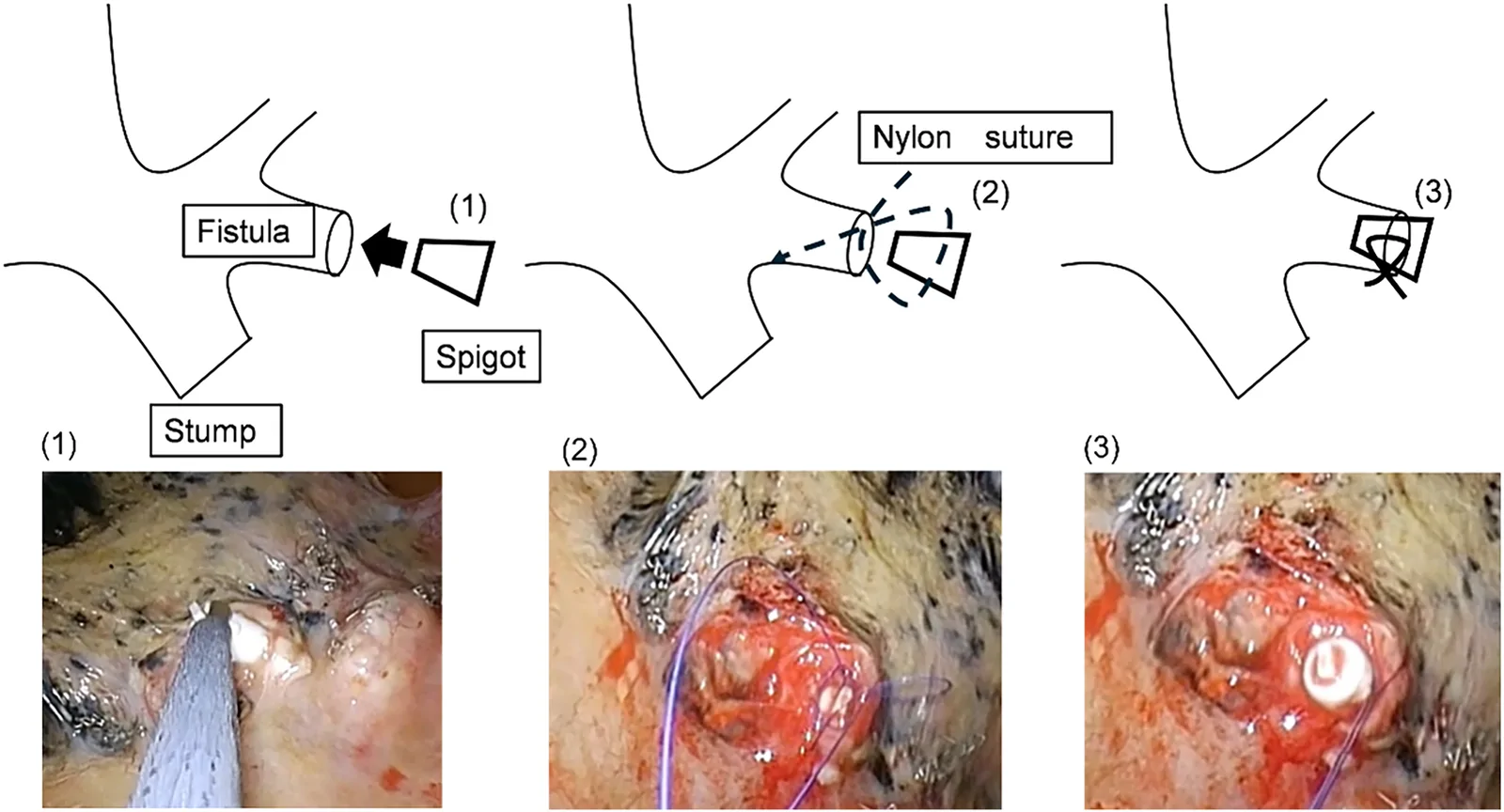
**Thoracoscopic suture fixation technique**. (1) The spigot was deployed into the fistula. (2) A suture was passed through the bronchial wall, the spigot, and the opposite bronchial wall. (3) The spigot was tied securely to the stump.

The drain insertion site was enlarged to 3 cm, allowing thoracoscopic visualization of the dislodged EWS, the remaining lung, and the bronchial fistula. Because direct suturing risked bronchial injury, the dislodged EWS was removed extracorporeally, and the bronchial wall was sutured in the following sequence: bronchial wall → EWS → bronchial wall, thereby fixing the EWS to the stump (Fig. 2, Video). A subsequent leak test revealed an additional pulmonary fistula separate from the main defect, which was cauterized. The procedure was completed with no residual air leak. The operative time was 1 hour and 38 minutes, and blood loss was 10 ml.

## 4 Results

Bacterial cultures detected *Stenotrophomonas maltophilia*, and a negative result was confirmed prior to surgery following oral administration of minocycline. The patient continued minocycline for one month postoperatively, and no recurrence has been observed after discontinuation. The chest drain was removed on postoperative day 3. Bronchoscopy at 6 months confirmed complete closure of the fistula (Fig. 1). The patient died 11 months later from recurrent colon cancer, but no recurrence of empyema or air leakage was observed. The patient's performance status remained at 1 before and immediately after surgery. After removal of the chest drain, the patient maintained a PS of 0 until symptoms related to recurrence developed.

## 5 Discussion

Postoperative bronchopleural fistula is difficult to manage, particularly when infection and tissue fragility make direct stump closure unsafe. The Endobronchial Watanabe Spigot (EWS) is widely used for persistent air leaks; however, in large fistulas, dislodgement or expectoration becomes a major limitation. Previous reports have described techniques such as traction-assisted placement to facilitate EWS insertion [3], but no method has been proposed to secure the device in large postoperative stump fistulas.

In empyema cases, the bronchial wall is often extremely fragile due to infection and inflammation, and primary stump suturing carries a high risk of tearing [3]. Direct suturing concentrates tension on the bronchial tissue, increasing the likelihood of rupture. By interposing the EWS between the suture bites, deformation of the bronchial wall was minimized and excessive tension on the tissue was avoided. The spigot acted as a buffer that distributed the suture load, allowing secure closure without risking bronchial tearing. Because the EWS is made of biocompatible silicone, granulation tissue gradually seals the surrounding space, enabling stable closure even when the device remains in situ.

Furthermore, bronchoscopic EWS placement provides only “deployment,” without the ability to fix the device. In large fistulas, this inherently unstable configuration leads to repeated dislodgement, as observed in our patient. In contrast, the thoracoscopic approach allows direct visualization of the fistula and true fixation by suturing, resulting in a more stable and durable occlusion. This difference is particularly important when the fistula exceeds the size of the largest available EWS.

Uniportal VATS was advantageous in this setting because it minimized surgical trauma and reduced the risk of further wound infection. The minimally invasive nature of the approach facilitated early recovery and allowed continuation of oncologic treatment. To our knowledge, this is the first report describing thoracoscopic suture fixation of an EWS to a postoperative bronchial stump.

## 6 Conclusion

This minimally invasive technique enabled secure closure of a large postoperative bronchial fistula that could not be controlled by bronchoscopic EWS placement alone. By suturing the EWS directly to the bronchial stump under thoracoscopic visualization, stable fixation was achieved without excessive tension on the fragile bronchial wall. This approach may serve as a useful option for managing large postoperative bronchial fistulas.

## CRediT authorship contribution statement

**Gaku Yamaguchi:** Writing – review & editing, Writing – original draft, Visualization, Validation, Supervision, Software, Resources, Project administration, Methodology, Investigation, Funding acquisition, Formal analysis, Data curation, Conceptualization. **Haruka Kanai:** Resources, Investigation. **Atsushi Morio:** Resources, Investigation.

## Informed consent

Written informed consent for publication was obtained from the patient.

## Data availability statement

Data are not publicly available due to privacy but may be provided on request.

## Ethics approval

This study was conducted under the institutional opt-out policy for thoracic malignancy–related clinical research, approved by the Institutional Review Board of the International University of Health and Welfare, Ichikawa Hospital (Protocol No. 18-Ic-77-②). The patient had provided general consent for the use of clinical information for research and educational purposes at the time of admission.

## Declaration of competing interests

The authors declare no conflicts of interest.
